# Supplementary material for: The 3D mutational constraint on amino acid sites in the human proteome
Source: Nat Commun. 2022 Jun 7;13:3273. doi: 10.1038/s41467-022-30936-x (PMC9174330; doi:10.1038/s41467-022-30936-x)
Supplement: Supplementary file 1 — Supplementary Information [file 41467_2022_30936_MOESM1_ESM.pdf]

Supplementary Figures

The 3D mutational constraint on amino acid sites in the human proteome

Bian Li [1,2,*], Dan M. Roden [2,3], John A. Capra [1,4,*]

1. Department of Biological Sciences, Vanderbilt University, Nashville, TN 37203, USA

2. Department of Medicine, Vanderbilt University Medical Center, Nashville, TN 37232, USA

3. Departments of Pharmacology and Biomedical Informatics, Vanderbilt University Medical Center, Nashville, TN 37232, USA

4. Bakar Computational Health Sciences Institute and Department of Epidemiology and Biostatistics, University of California, San Francisco, CA 94143, USA

* To whom correspondence should be addressed: bian.li@vanderbilt.edu, tony@capralab.org

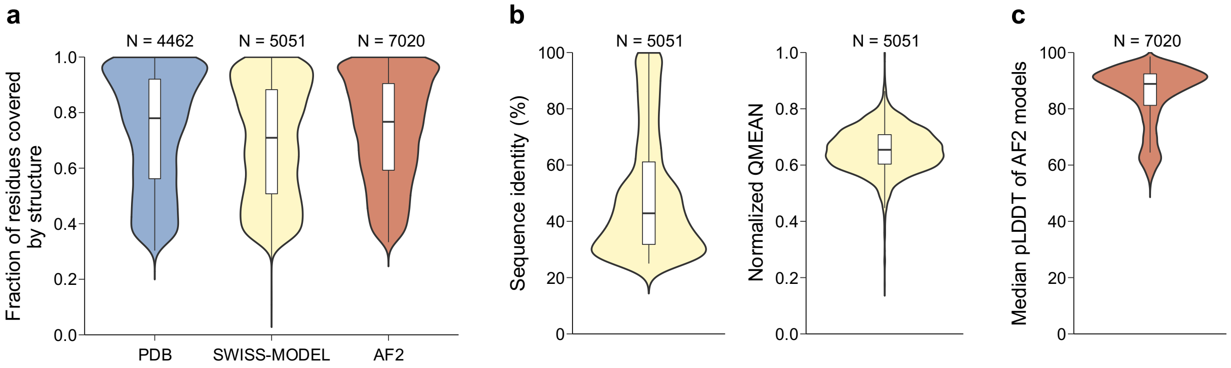

Supplementary Figure 1. Coverage and quality of protein 3D structures used in COSMIS. a) Distribution of the fraction of residues covered by 3D structures for all proteins in the human reference proteome for which COSMIS scores are computed. Distributions are plotted separately for each of the three protein 3D structure sources. Median sequence coverage of structures from PDB, SWISS-MODEL, and AF2 are 78.0%, 70.9%, and 76.7%, respectively. **b**) Left: Distribution of sequence identity of SWISS-MODEL homology models (median = 42.9%); Right: Distribution of normalized QMEAN scores in the range [0, 1] of SWISS-MODEL homology models (median = 0.654). **c**) For each AlphFold2 (AF2) protein 3D structure model used in COSMIS, we computed the median pLDDT score in the range [0, 100] of the residues for which COSMIS scores were calculated. The plot shows the distribution of median pLDDT scores of 7,020 AF2 models (median = 88.8). In boxplot graphs centre line indicates median, bounds of box indicate 25th and 75th percentiles, and whiskers indicate minimum and maximum. PDB: Protein Data Bank; AF2: AlphaFold2; pLDDT: per-residue local distance difference test. Source data are provided as a Source Data file.

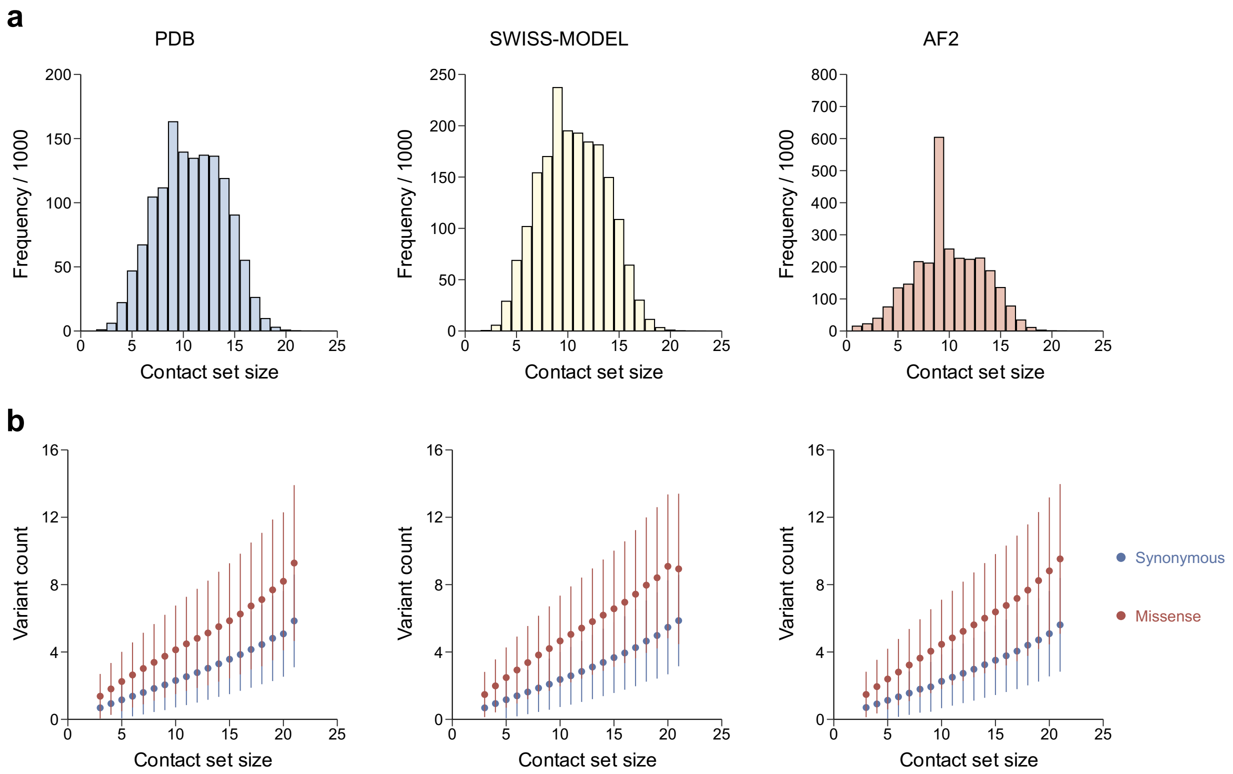

Supplementary Figure 2. Statistics of contact sets computed based on structures from PDB, SWISS-MODEL, and AF2, respectively. a) Distributions of the size of contact set (number of amino acid sites in the contact set). The overall distributions are similar across difference protein 3D structure sources. **b**) Distribution of synonymous and missense variant counts across different sizes of contact set. Again, the overall distributions are similar regardless of the source of protein 3D structures. Error bars indicate standard deviations. Precise sample size values were reported in Supplementary Data 16. Source data are provided as a Source Data file.

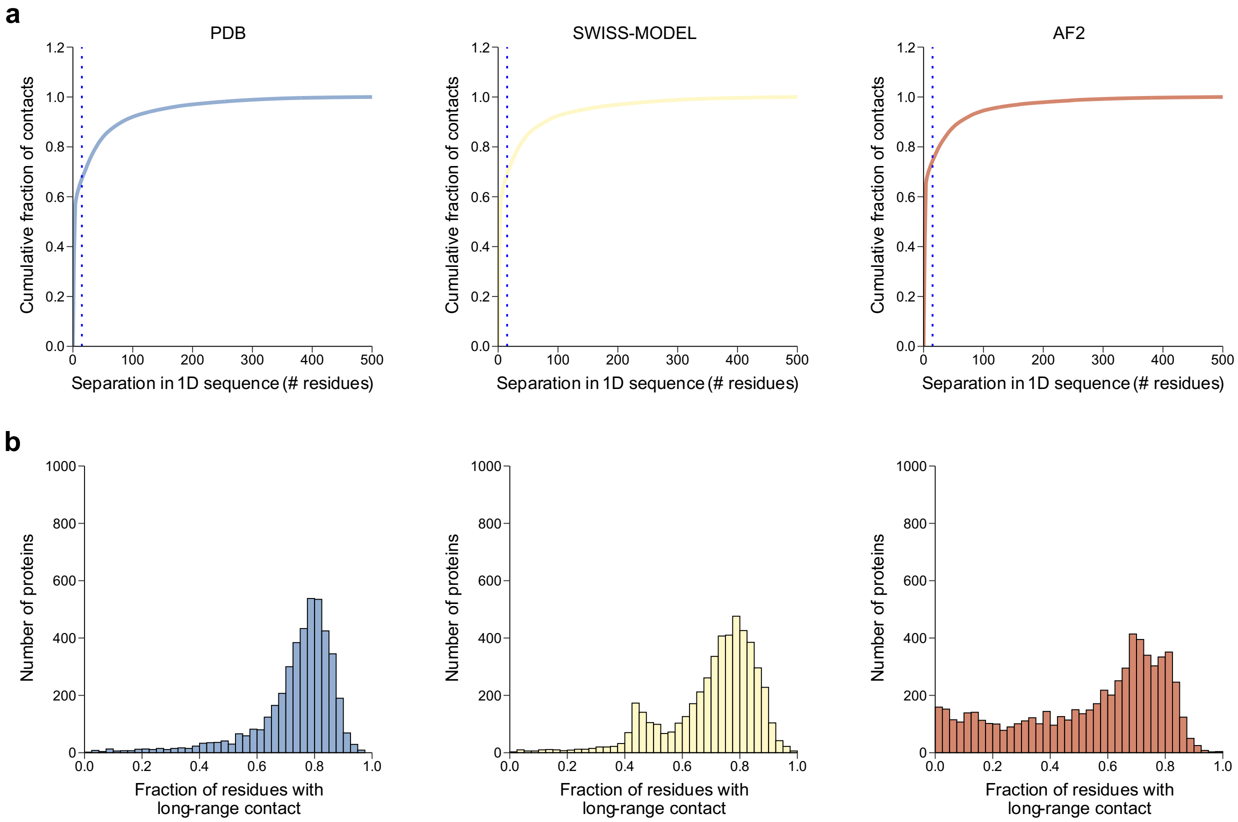

Supplementary Figure 3. Statistics on long-range contacts observed in protein 3D structures from the PDB, SWISS-MODEL, and AF2 databases. a) Cumulative distribution of the sequence separation (number of residues apart in sequence) of all 3D contacts observed in structures from the three protein structure databases, respectively. Blue dotted lines correspond to a separation of 15 residues in 1D sequence. **b**) Distributions of per-protein fraction of residues that make at least one long-range contact. PDB: Protein Data Bank; AF2: AlphaFold2. Source data are provided as a Source Data file.

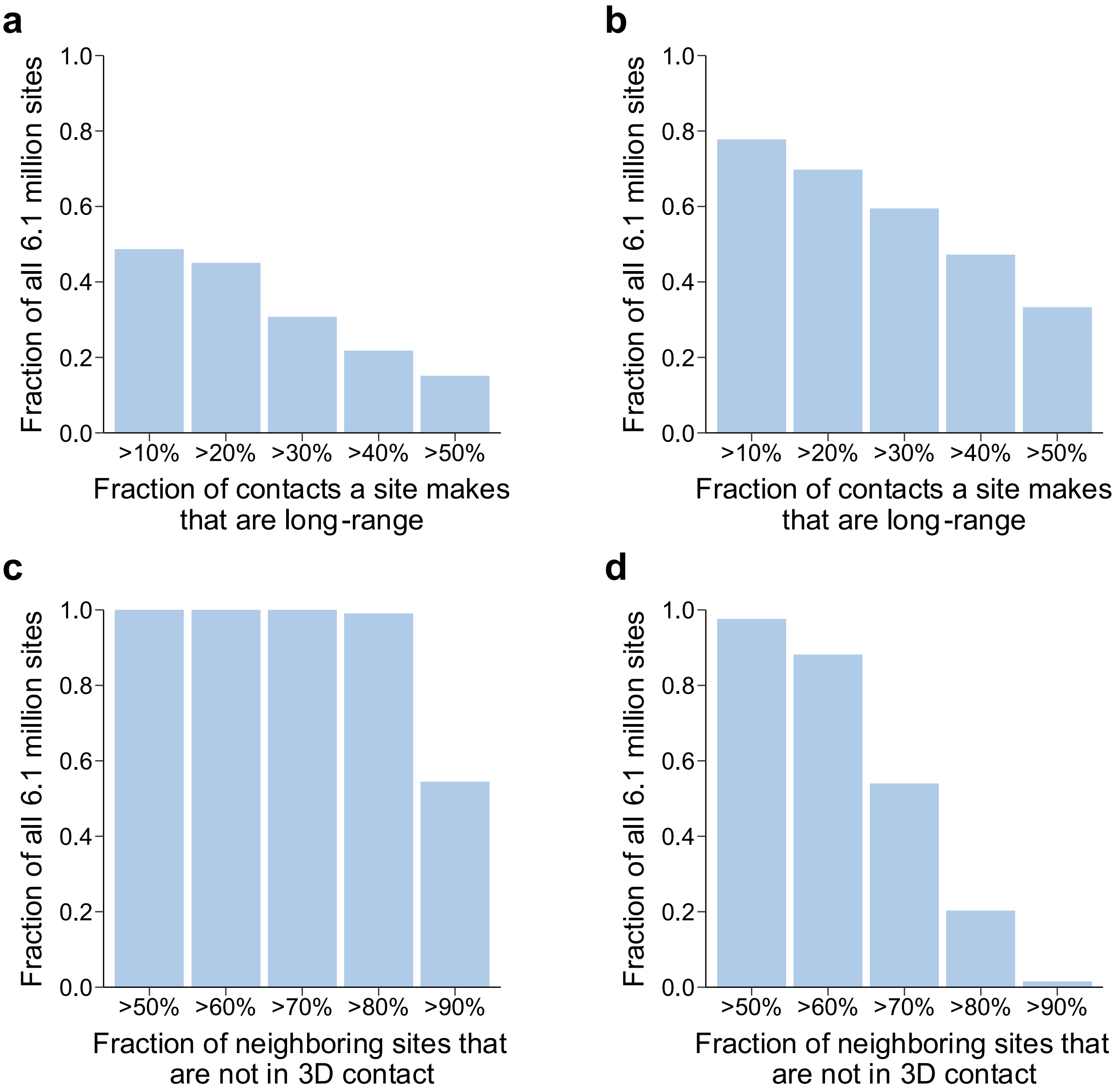

Supplementary Figure 4. Statistics of long-range contacts (separated by more than 15 residues along the 1D sequence) at 6 Å and 10 Å distance thresholds. a, **b**) Contact sets capture long-range sites that interact in 3D at 6 Å and 10 Å distance thresholds, respectively. The bar plots show the fraction of all 6.1 million sites with at least a certain fraction of long-range 3D contacts in their contact sets. **c**, **d**) Similar to Fig. 2d, but at 6 Å and 10 Å distance thresholds, respectively. The bar plots show the fraction of all 6.1 million sites that have at least a certain fraction of 1D sequence neighbors that do not form 3D contacts. Source data are provided as a Source Data file.

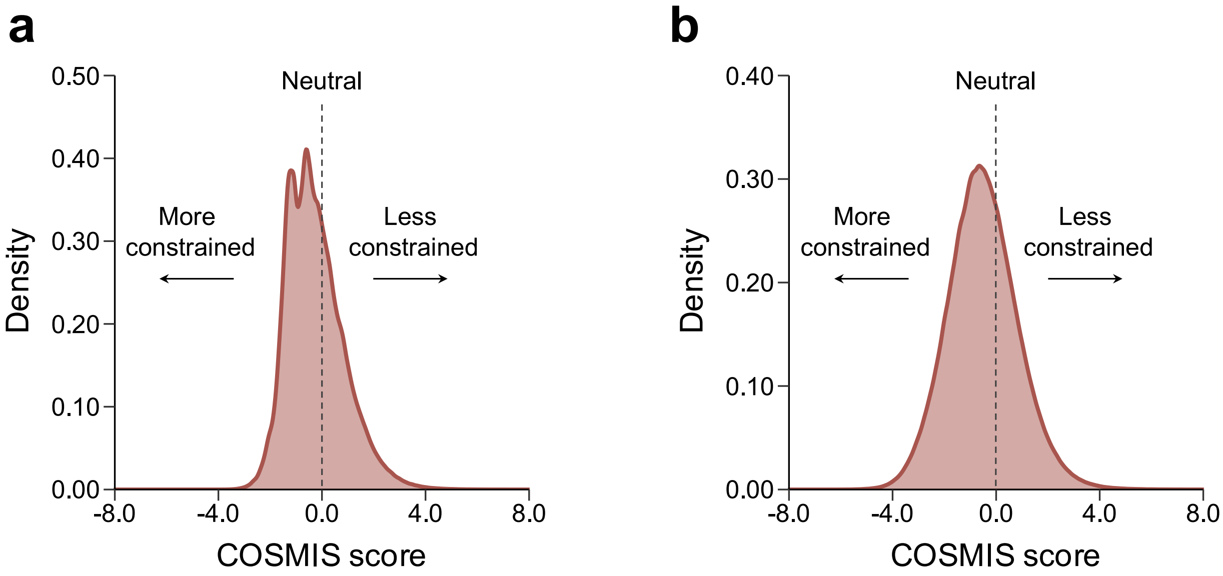

Supplementary Figure 5. Distribution of the COSMIS scores for 6.1 million unique amino acid sites of the reference human proteome, computed at 6 Å (a) and 10 Å (b) distance thresholds, respectively. As with the score distribution computed at 8 Å distance threshold, an average amino acid site in the human proteome is depleted of missense variants in its contact set (median COSMIS scores are -0.498 and -0.554 at 6 Å and 8 Å distance thresholds, respectively). Source data are provided as a Source Data file.

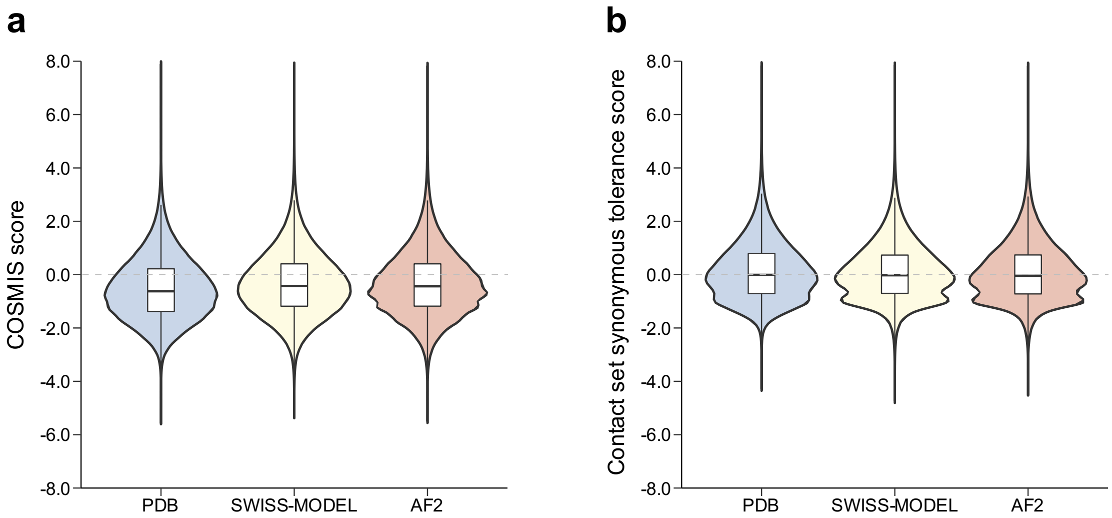

Supplementary Figure 6. Distributions of COSMIS (COntact Set MISsense tolerance) and contact set synonymous tolerance scores by sources of protein 3D structures. a) Distributions of COSMIS scores computed based on difference sources of protein 3D structures. In general, proteins with experimentally determined structures in the PDB have a significantly lower COSMIS score than those that only have computationally predicted structures in SWISS-MODEL or AF2 databases (median -0.62 vs. -0.42 and 0.44, respectively, $p < 2.2 \times 10^{-308}$, two-sided Mann-Whitney U test). **b**) Distributions of contact set synonymous tolerance score computed based on difference sources of protein 3D structures. The scores are centered at the expected score under neutrality (i.e., median 0) regardless of the sources of protein 3D structures, consistent with the hypothesis that synonymous variants are not subject to 3D spatial constraint in protein structures. In boxplot graphs centre line indicates median, bounds of box indicate 25th and 75th percentiles, and whiskers indicate minimum and maximum. n=1,373,513; 1,888,636; 2,848,473 unique amino acid sites for PDB, SWISS-MODEL, and AF2, respectively. PDB: Protein Data Bank; AF2: AlphaFold2. Source data are provided as a Source Data file.

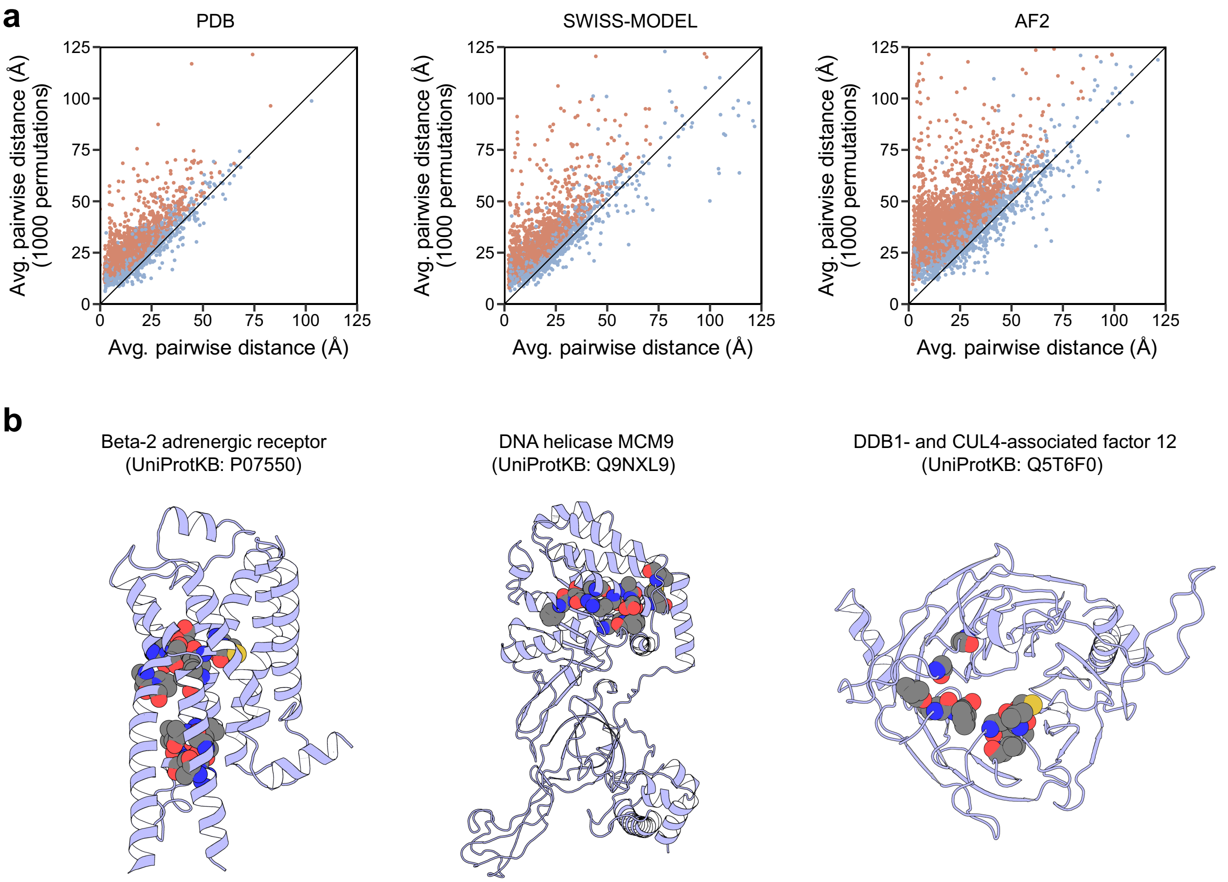

Supplementary Figure 7. High-confidence constrained sites in most proteins are clustered. a) To determine whether the set of high-confidence constrained sites of a protein are clustered, we compare the average pairwise distance between these sites with that expected if the same number of sites were chosen randomly. We derived the expected average pairwise distance through 1,000 permutations based on the same structure used to compute the COSMIS scores. Dots above the diagonal represent proteins whose high-confidence sites are clustered. Those with an empirical p value < 0.01 are colored in salmon red. The median ratio of average pairwise distance between high-confidence sites compared to that expected from permutations is 0.66. **b**) Examples of the clustering of high-confidence sites in three proteins. Residues at high-confidence sites are rendered in spheres colored according to element types (dark gray: carbon, blue: nitrogen, red: oxygen, yellow: sulfur). PDB: Protein Data Bank; AF2: AlphaFold2. Source data are provided as a Source Data file.

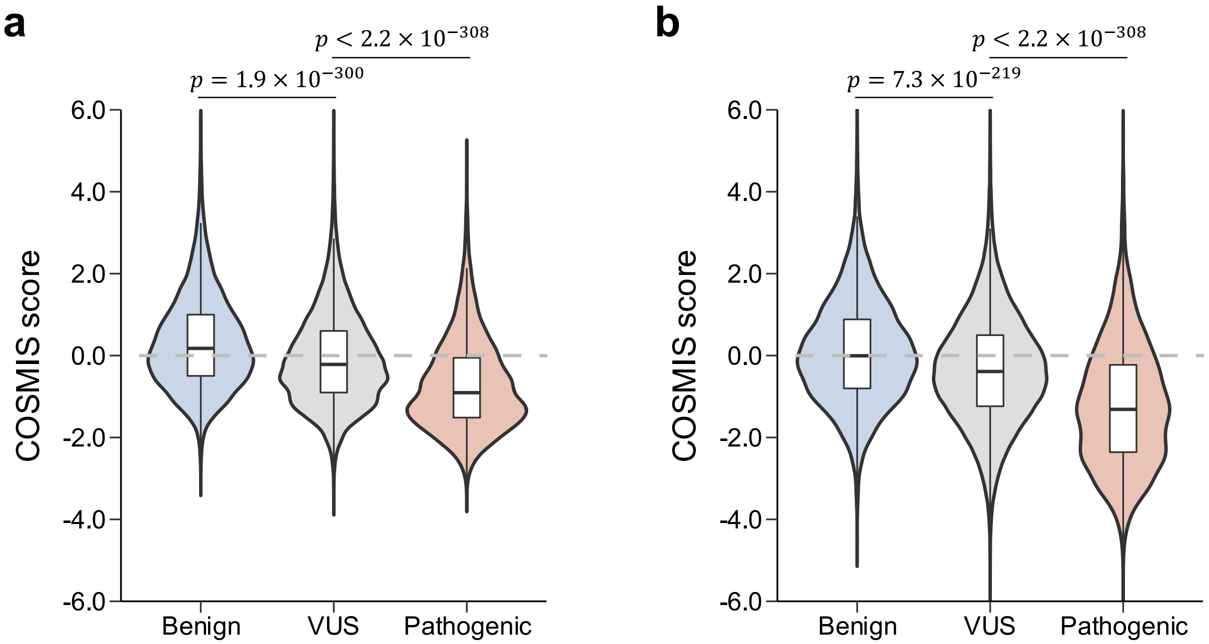

Supplementary Figure 8. Distributions of COSMIS scores computed at 6 Å (a) and 10 Å (b) for ClinVar variant sets. a) Median scores of benign, VUS, and pathogenic variants using a 6 Å threshold to define contact sets are 0.10, -0.22, and -0.87, respectively. **a**) Median scores of benign, VUS, and pathogenic variants using a 10 Å threshold to define contact sets are -0.10, -0.39, and -1.29, respectively. All p values were computed from two-sided Mann Whitney U tests. In boxplot graphs centre line indicates median, bounds of box indicate 25th and 75th percentiles, and whiskers indicate minimum and maximum. VUS: variants of uncertain significance. Source data are provided as a Source Data file.

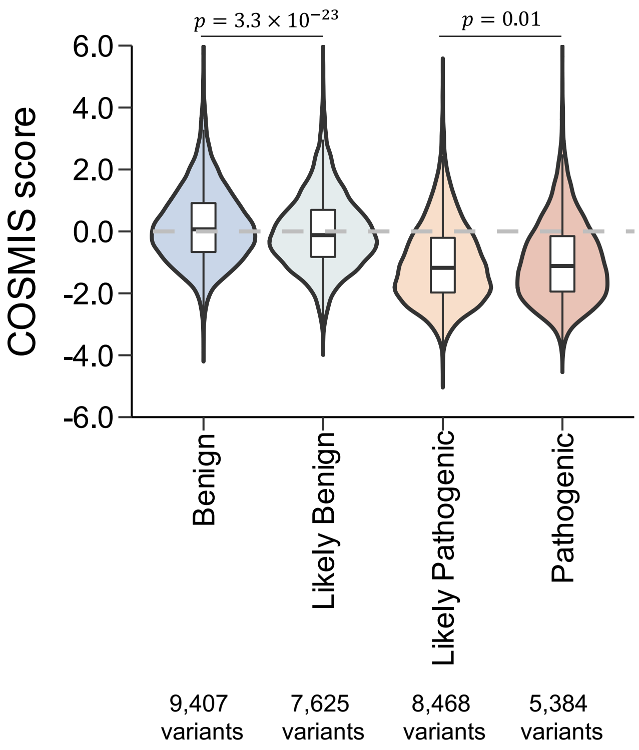

Supplementary Figure 9. Distributions of COSMIS scores for subgroups of variants from ClinVar. Further division of variants into four subgroups, i.e., benign, likely benign, likely pathogenic, and pathogenic, shows that the median score of likely benign variants is slightly lower than that of benign variants (-0.12 vs. 0.07; $p = 3.3 \times 10^{-23}$, two-sided Mann-Whitney U test), whereas little difference exists between pathogenic and likely pathogenic variants (median -1.12 vs. -1.17, respectively; $p = 0.01$, two-sided Mann-Whitney U test). In boxplot graphs centre line indicates median, bounds of box indicate 25th and 75th percentiles, and whiskers indicate minimum and maximum. Source data are provided as a Source Data file.

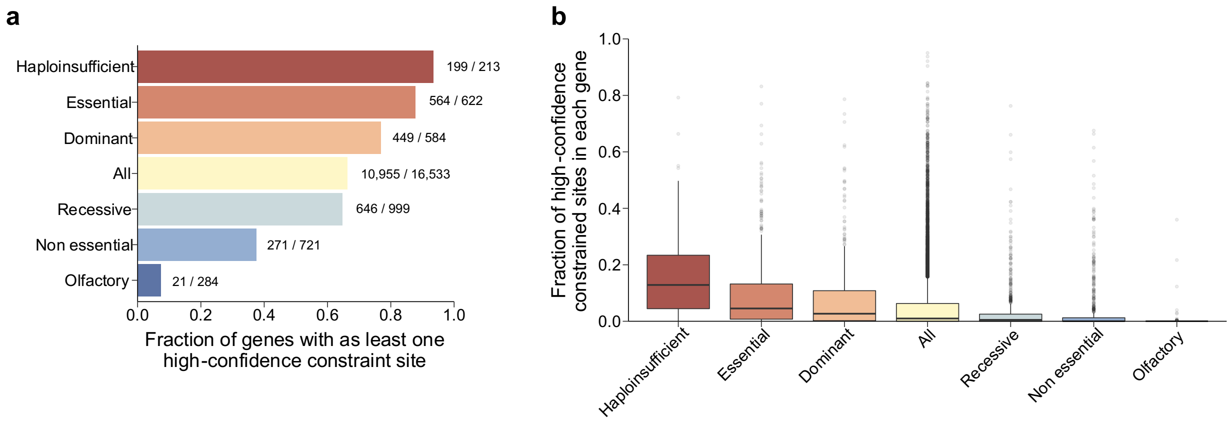

Supplementary Figure 10. Abundance of high-confidence constrained sites in genes with different levels of functional constraint. a) Fraction of genes with at least one high-confidence constrained site in each of the six categories of genes. **b**) Distribution of per-gene fraction of high-confidence constrained amino acid sites of each gene category. In general, high-confidence constrained sites are more common in genes with essential functions and disease associations compared to genes with functions less essential to health and fitness. In boxplot graphs center line indicates median, bounds of box indicate 25th and 75th percentiles, and whiskers indicate minimum and maximum. Sample size values are the same as in panel a and Fig. 5c. Source data are provided as a Source Data file.

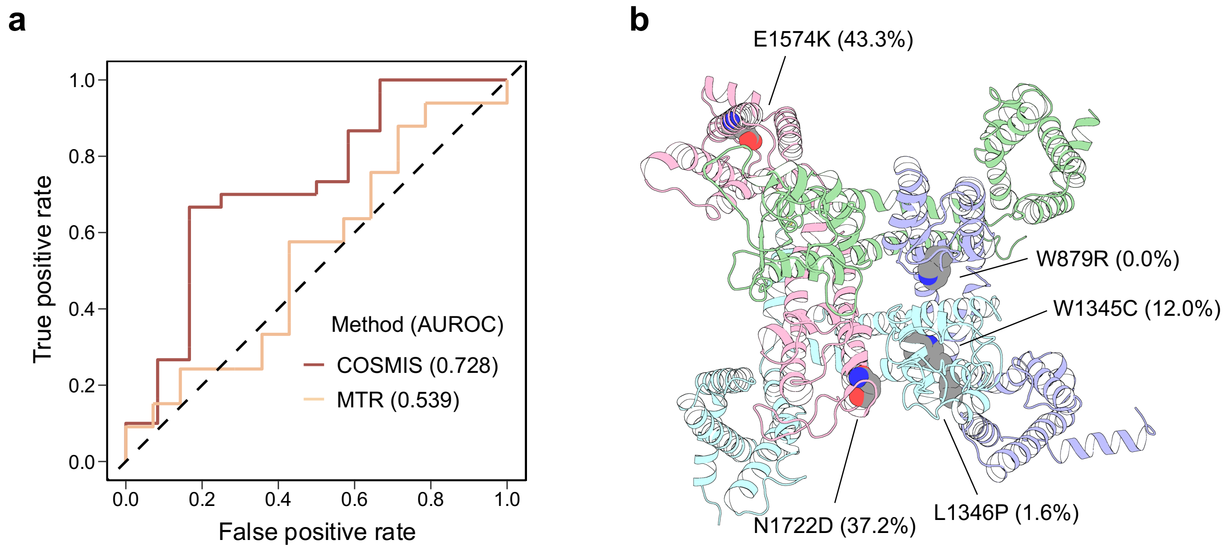

Supplementary Figure 11. Applying COSMIS to classify the pathogenicity of SCN5A variants of uncertain significance (VUS). a) To demonstrate the utility of COSMIS in real-world interpretation of VUS, we computed the ROC curves of COSMIS and MTR based on the 44 SCN5A VUS reclassified in Glazer *et al.* using a high-throughput functional assay (12 benign/likely benign and 32 pathogenic/likely pathogenic; Supplementary Data 7). COSMIS performs substantially better than MTR, illustrating the benefits of incorporating 3D structural context. **b**) Our analysis further highlights five SCN5A VUS (W879R, W1345C, L1346P, E1574K, and N1722D) that are ranked among the top 10 variants most likely to be pathogenic by COSMIS. None of these variants is ranked among the top 10 by MTR (Supplementary Data 7). These VUS were classified as likely to be pathogenic according to functional data reported in Glazer *et al.* (peak current density shown as % of wild type in parentheses). The SCN5A structure is rendered in cartoon and colored by domain (PDB ID: 6LQA [http://doi.org/10.2210/pdb6LQA/pdb]). Sites where these five variants are located are rendered in spheres. AUROC: area under the receiver operating characteristic. Source data are provided as a Source Data file.

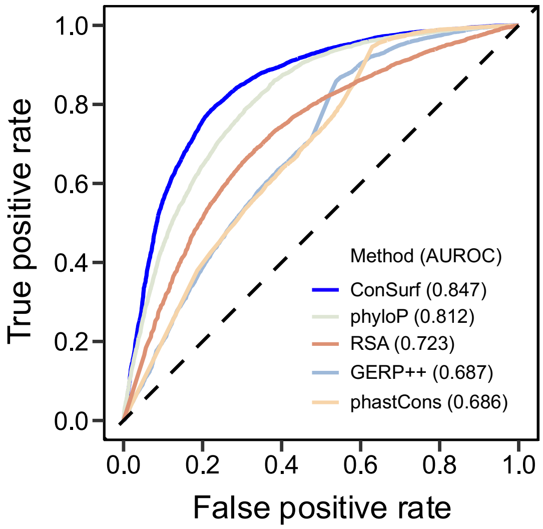

Supplementary Figure 12. Performance of phylogenetic conservation metrics in predicting variant pathogenicity. The evaluation was performed on a total of 8,062 benign and 7,256 pathogenic missense variants from ClinVar for which all scores can be computed (Supplementary Data 6). We also evaluated the performance of relative solvent accessibility (RSA) in addition to four phylogenetic conservation metrics (GERP++, phyloP, phastCons, and ConSurf). Here, the best-performing metric is ConSurf. Note that in contrast to ConSurf, GERP++, phyloP, and phastCons quantify constraint on nucleotide sequence. AUROC: area under the receiver operating characteristic. Source data are provided as a Source Data file.

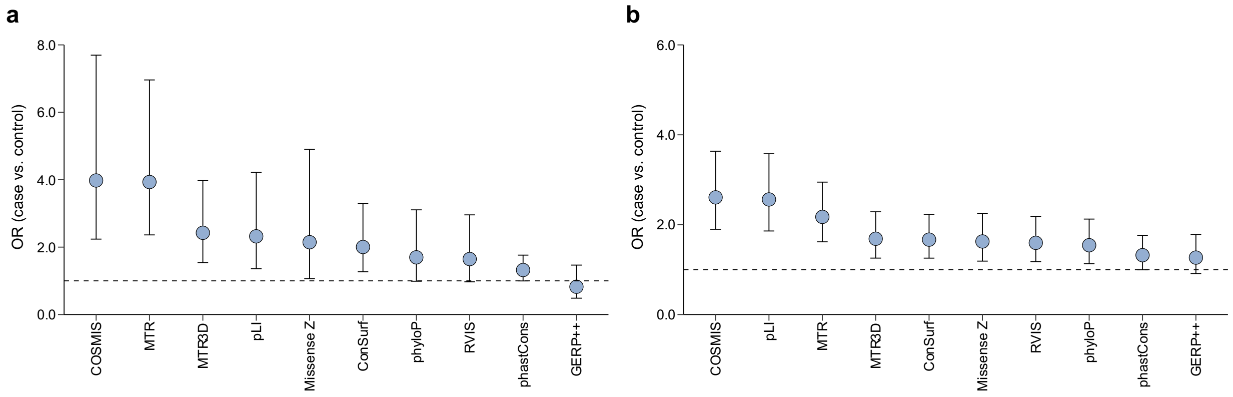

Supplementary Figure 13. Case variant enrichment analysis for intra- and inter-species constraint metrics at additional thresholds. a) ORs of evaluated constraint metrics at the 5th percentile most constrained sites. COSMIS has the highest enrichment for cases (OR 4.0, 95% confidence interval [2.2, 7.7]). **b**) ORs of evaluated constraint metrics at the 20th percentile most constrained sites. While the ORs at 20th percentile threshold are generally lower than at 5th and 10th percentile thresholds, COSMIS still has the highest enrichment for cases (OR 2.6, 95% confidence interval [1.9, 3.6]) compared to all other evaluated metrics. Error bars are 95% confidence intervals of ORs. The values for each cell of the contingency table used for the OR calculation in each percentile bin were reported in Supplementary Data 15. OR: odds ratio. Source data are provided as a Source Data file.

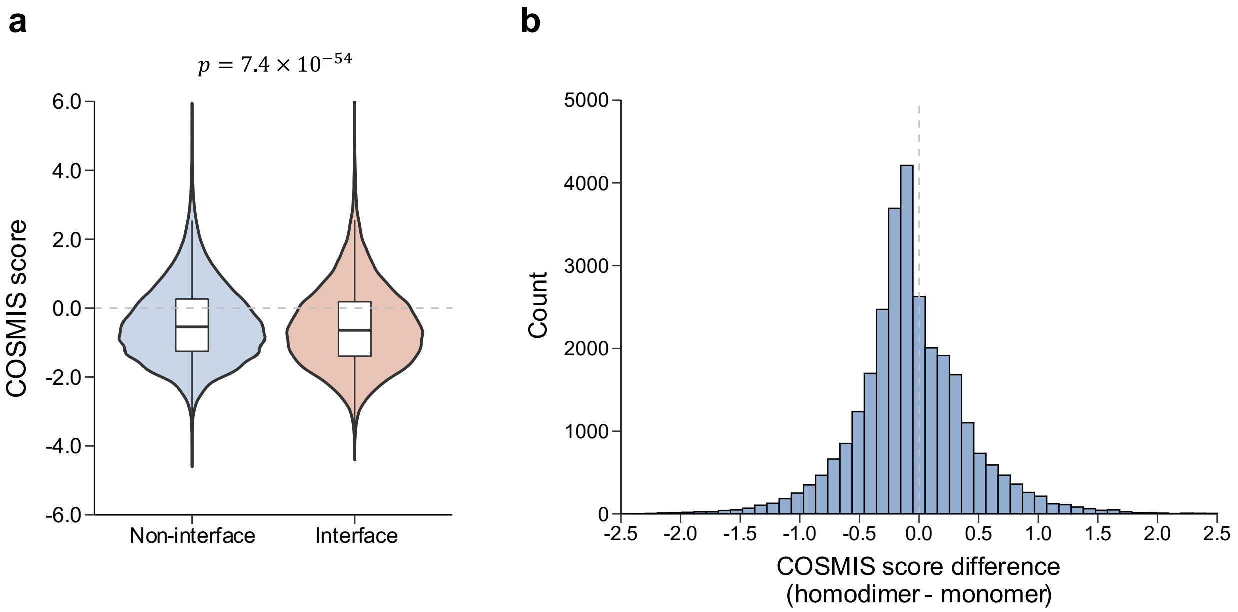

Supplementary Figure 14. Applying COSMIS to homodimeric structures. **a**) COSMIS score distributions for interface and non-interface amino acid sites in 1,678 homodimeric protein structures from the PDB. Overall, interface sites involved in oligomerization (making more 3D contacts in oligomers than in monomers) have significantly lower COSMIS scores than non-interface sites (median -0.64 vs. -0.55, $p = 7.4 \times 10^{-54}$, two-sided Mann Whitney U test). In boxplot graphs center line indicates median, bounds of box indicate 25th and 75th percentiles, and whiskers indicate minimum and maximum. **b**) COSMIS scores of interface sites computed based on homodimers are generally lower than those computed based on monomers (median difference -0.11, $p = 3.1 \times 10^{-12}$, two-sided Mann-Whitney U test). Source data are provided as a Source Data file.

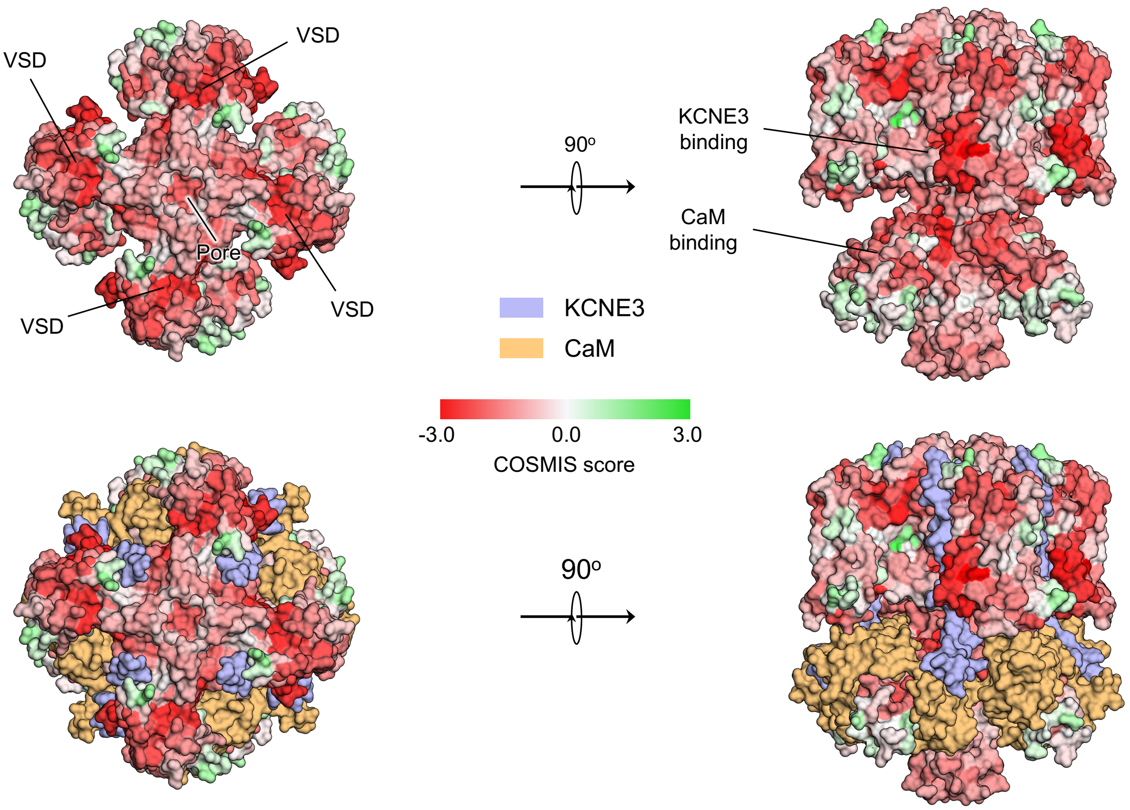

Supplementary Figure 15. Mapping of COSMIS scores of residues of the KCNQ1 tetrameric potassium channel to structure highlights the clustering of functionally important regions. The VSD and the pore, which are responsible for sensing the changes in membrane potential and conducting potassium ion flow respectively, stand out as strongly constrained (more negative COSMIS scores) regions in KCNQ1. Regions that bind KCNE3 and CaM, which are believed to be essential regulators of KCNQ1 function (Sun and MacKinnon, 2020), are also strongly constrained. PDB ID of the structure: 6V01 [http://doi.org/10.2210/pdb6V01/pdb]. VSD: voltage-sensing domain; CaM: calmodulin. Source data are provided as a Source Data file.

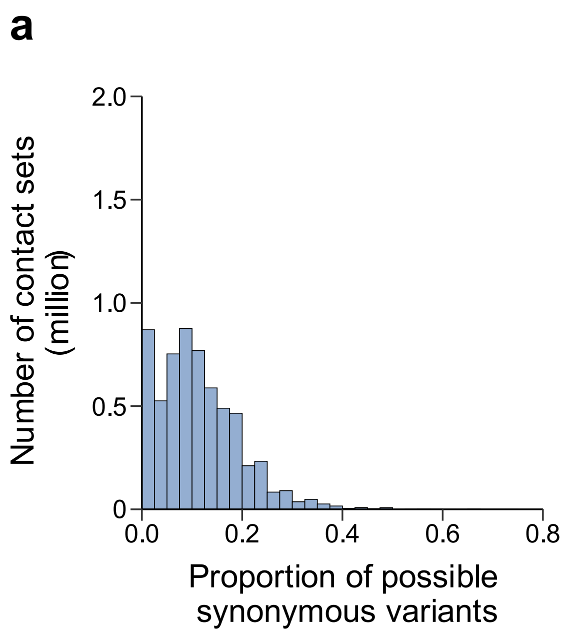

Supplementary Figure 16. Proportion of all possible a) synonymous and b) missense variants observed in each contact set. On average, 10.3% and 6.3% of all possible synonymous and missense variants in a contact set are observed in gnomAD, respectively. Source data are provided as a Source Data file.

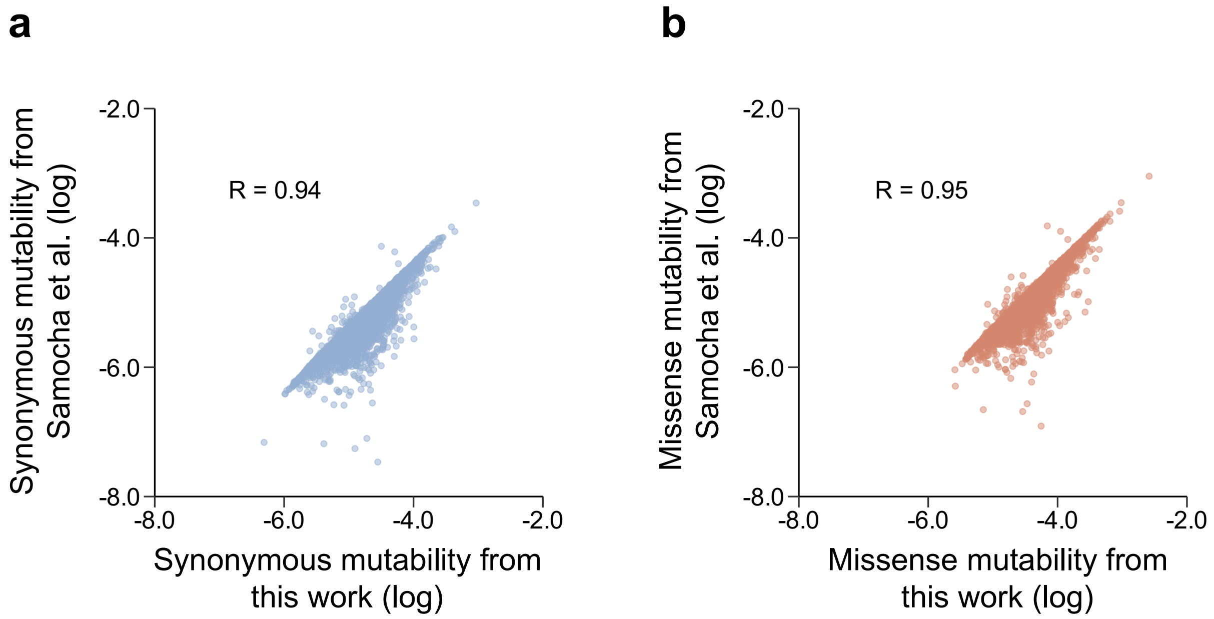

Supplementary Figure 17. Agreement between estimates of per-protein total synonymous and missense mutability used in COSMIS and previous work. The scatter plots and Pearson's R are both based on the total synonymous and missense mutability of 14,756 Ensembl canonical transcripts with estimates from Samocha et al. 2014 (see main text for the reference to the paper). Source data are provided as a Source Data file.

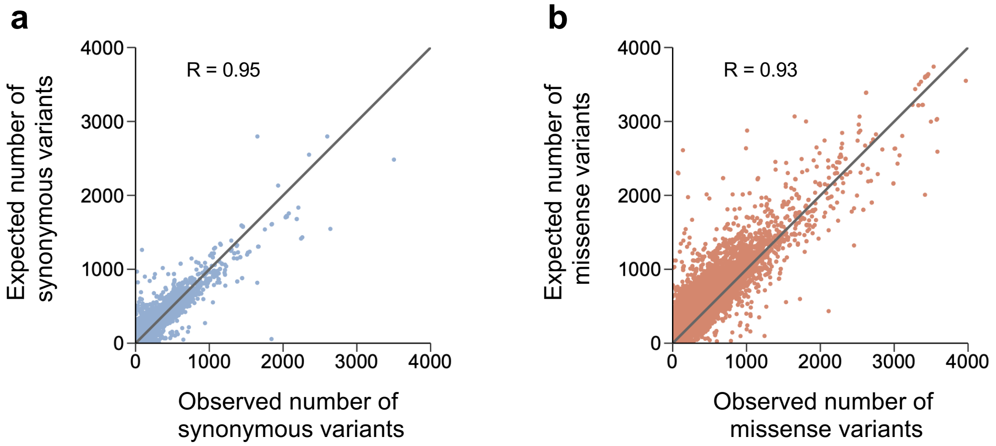

Supplementary Figure 18. Agreement between per-protein total number of observed and expected synonymous and missense variants. The total expected number of variants per protein was computed based on the relation between synonymous variant count and synonymous mutability (from the fitted linear regression), i.e., $\hat{y} = 6.42 \times 10^6 \times \mu - 0.18$, where μ is per-protein total synonymous or missense mutability. Source data are provided as a Source Data file.
