# Supplementary material for: The 3D mutational constraint on amino acid sites in the human proteome
Source: Nat Commun. 2022 Jun 7;13:3273. doi: 10.1038/s41467-022-30936-x (PMC9174330; doi:10.1038/s41467-022-30936-x)
Supplement: Supplementary file 3 — Description of additional Supplementary File [file 41467_2022_30936_MOESM3_ESM.pdf]

Descriptions of additional supplementary Data Files

Supplementary Data 1. Summary of proteins in the human proteome for which COSMIS scores have been computed.

Supplementary Data 2. High-confidence constrained sites in the human proteome.

Supplementary Data 3. COSMIS scores of unambiguously annotated ClinVar variants.

Supplementary Data 4. ClinVar pathogenic variants of proteins with at least one high-confidence site.

Supplementary Data 5. Extremely constrained proteins identified according to fraction of high-confidence sites.

Supplementary Data 6. Interspecies conservation and intra-human constraint scores of CinVar variants.

Supplementary Data 7. COSMIS and MTR scores of a set of functionally characterized VUS in the SCN5A sodium channel.

Supplementary Data 8. COSMIS scores of de novo variants.

Supplementary Data 9. Interspecies conservation and intra-human constraint scores of de novo variants.

Supplementary Data 10. Summary of potassium ion channel structure models.

Supplementary Data 11. Per-transcript total synonymous mutability and missense mutability.

Supplementary Data 12. Sources of interspecies conservation and intra-human constraint scores.

Supplementary Data 13. Homodimer proteins obtained from the INSIDER resource.

Supplementary Data 14. The values for each cell of the contingency table used for the odds ratio (pathogenic vs. benign) calculation in each COSMIS percentile bin.

Supplementary Data 15. The values for each cell of the contingency table used for the odds ratio (case vs. control) calculation in the 5th, 10th, and 20th percentiles of the scores.

Supplementary Data 16. Precise sample size values and variant count statistics at each contact set size.
