# Supplementary material for: The 3D mutational constraint on amino acid sites in the human proteome
Source: Nat Commun. 2022 Jun 7;13:3273. doi: 10.1038/s41467-022-30936-x (PMC9174330; doi:10.1038/s41467-022-30936-x)
Supplement: Supplementary file 5 — Reporting Summary [file 41467_2022_30936_MOESM5_ESM.pdf]

nature portfolio

Corresponding author(s): Bian Li and John A. Capra

Last updated by author(s): May 12, 2022

Reporting Summary

Nature Portfolio wishes to improve the reproducibility of the work that we publish. This form provides structure for consistency and transparency in reporting. For further information on Nature Portfolio policies, see our Editorial Policies and the Editorial Policy Checklist.

Statistics

For all statistical analyses, confirm that the following items are present in the figure legend, table legend, main text, or Methods section.

n/a	Confirmed	
☐	☒	The exact sample size (*n*) for each experimental group/condition, given as a discrete number and unit of measurement
☒	☐	A statement on whether measurements were taken from distinct samples or whether the same sample was measured repeatedly
☐	☒	The statistical test(s) used AND whether they are one- or two-sided<br>*Only common tests should be described solely by name; describe more complex techniques in the Methods section.*
☒	☐	A description of all covariates tested
☒	☐	A description of any assumptions or corrections, such as tests of normality and adjustment for multiple comparisons
☐	☒	A full description of the statistical parameters including central tendency (e.g. means) or other basic estimates (e.g. regression coefficient) AND variation (e.g. standard deviation) or associated estimates of uncertainty (e.g. confidence intervals)
☐	☒	For null hypothesis testing, the test statistic (e.g. *F*, *t*, *r*) with confidence intervals, effect sizes, degrees of freedom and *P* value noted<br>*Give P values as exact values whenever suitable.*
☒	☐	For Bayesian analysis, information on the choice of priors and Markov chain Monte Carlo settings
☒	☐	For hierarchical and complex designs, identification of the appropriate level for tests and full reporting of outcomes
☐	☒	Estimates of effect sizes (e.g. Cohen's *d*, Pearson's *r*), indicating how they were calculated

Our web collection on statistics for biologists contains articles on many of the points above.

Software and code

Policy information about availability of computer code

Data collection	Biopython 1.80.dev0<br>NumPy 1.20.3<br>Open-Source PyMOL 2.5.0<br>pandas 1.1.2<br>plotROC 2.2.1<br>PNWColors release 1<br>Python 3.7.9<br>R 4.0.2<br>ROCR 1.0-11<br>Rate4site 2.0.1<br>Tidyverse 1.3.1<br>VEP release 96.0
Data analysis	A custom code was developed as part of the analysis in this work. The code base is fully open on the code sharing platform GitHub at https://github.com/CapraLab/cosmis.

For manuscripts utilizing custom algorithms or software that are central to the research but not yet described in published literature, software must be made available to editors and reviewers. We strongly encourage code deposition in a community repository (e.g. GitHub). See the Nature Portfolio guidelines for submitting code & software for further information.

Data

Policy information about availability of data

All manuscripts must include a data availability statement. This statement should provide the following information, where applicable:

- Accession codes, unique identifiers, or web links for publicly available datasets
- A description of any restrictions on data availability
- For clinical datasets or third party data, please ensure that the statement adheres to our policy

The code and source data for reproducing all graphs in the main text figures and supplementary figures (except figure 1, which is a schematic with no source data) are provided with this paper in the Source Data file [https://figshare.com/articles/dataset/Source_Data/19742404]. Precomputed COSMIS scores for 16,533 proteins from the UniProt human reference proteome can be downloaded at https://github.com/CapraLab/cosmis.

The following publicly available datasets and databases were used:
1000 Genomes phase 3: http://ftp.1000genomes.ebi.ac.uk/vol1/ftp/release/20130502/
AlphaFold2: https://ftp.ebi.ac.uk/pub/databases/alphafold/latest/UP000005640_9606_HUMAN_v2.tar
ClinVar (retrieved in August 2021): https://ftp.ncbi.nlm.nih.gov/pub/clinvar/vcf_GRCh38/
GECODE (release 34): http://ftp.ebi.ac.uk/pub/databases/gencode/Gencode_human/
Gene lists: https://github.com/macarthur-lab/gene_lists
gnomAD v2.1.1: https://gnomad.broadinstitute.org/
INSIDER (accessed in Jan. 2022): http://interactomeinsider.yulab.org
PDB: https://www.rcsb.org/
SIFTS: https://www.ebi.ac.uk/pdbe/docs/sifts/quick.html
SWISS-MODEL: https://swissmodel.expasy.org/repository/species/9606
UniProt: https://www.uniprot.org/help/uniprotkb
UniProt human reference proteome (release 2021_03): https://www.uniprot.org/proteomes/UP000005640

Field-specific reporting

Please select the one below that is the best fit for your research. If you are not sure, read the appropriate sections before making your selection.

[x] Life sciences [ ] Behavioural & social sciences [ ] Ecological, evolutionary & environmental sciences

For a reference copy of the document with all sections, see nature.com/documents/nr-reporting-summary-flat.pdf

Life sciences study design

All studies must disclose on these points even when the disclosure is negative.

Sample size	This study only used published datasets from prior studies. No sample size calculation was involved. The maximum number of samples available in these datasets were used whenever possible.
Data exclusions	No data exclusions.
Replication	We have run the framework at least three times, producing consistent results.
Randomization	Not applicable. This work did not involve assignment of subjects to experimental and control groups. Randomization is not generally used in this field.
Blinding	Not applicable. This work did not involve assignment of subjects to experimental and control groups. Blinding is not generally used in this field.

Reporting for specific materials, systems and methods

We require information from authors about some types of materials, experimental systems and methods used in many studies. Here, indicate whether each material, system or method listed is relevant to your study. If you are not sure if a list item applies to your research, read the appropriate section before selecting a response.

Materials & experimental systems

n/a	Involved in the study	
☒	☐	Antibodies
☒	☐	Eukaryotic cell lines
☒	☐	Palaeontology and archaeology
☒	☐	Animals and other organisms
☒	☐	Human research participants
☒	☐	Clinical data
☒	☐	Dual use research of concern

Methods

n/a	Involved in the study	
☒	☐	ChIP-seq
☒	☐	Flow cytometry
☒	☐	MRI-based neuroimaging
